# A recursive bifurcation model for early forecasting of COVID-19 virus spread in South Korea and Germany

**DOI:** 10.1038/s41598-020-77457-5

**Published:** 2020-11-27

**Authors:** Julia Shen

**Affiliations:** Dartmouth College, Hanover, NH USA

**Keywords:** Diseases, Health care

## Abstract

Early forecasting of COVID-19 virus spread is crucial to decision making on lockdown or closure of cities, states or countries. In this paper we design a recursive bifurcation model for analyzing COVID-19 virus spread in different countries. The bifurcation facilitates recursive processing of infected population through linear least-squares fitting. In addition, a nonlinear least-squares fitting procedure is utilized to predict the future values of infected populations. Numerical results on the data from two countries (South Korea and Germany) indicate the effectiveness of our approach, compared to a logistic growth model and a Richards model in the context of early forecast. The limitation of our approach and future research are also mentioned at the end of this paper.

## Introduction

Coronavirus disease (COVID-19) is a novel respiratory illness that originated in 2019 and can spread from person to person, as defined by Centers for Disease Control and Prevention (CDC)^1^. The first incidence of such disease was publicly reported to World Health Organization (WHO) as an outbreak in Wuhan, China, on 31 December 2019^2,3^. It was assumed on an association with the consumption of wild animals sold at Huanan Seafood Wholesale Market^4,5^. So far, the original source of this disease has not been clearly identified and the disease is continuously spread in over 70 countries. Reverse polymerase chain reactions and genome sequencing were used for diagnostics and therapeutics measures^6^. COVID-19 is a member of coronavirus family, and it is contagious among humans and animals^7^. Coronaviruses are a group of RNA viruses; the earliest study on animal coronavirus was reported in the late 1920s^8^, and human coronavirus was first studied by Kendall, Bynoe and Tyrell in 1960s through extracting the viruses from patients who suffered from common colds^9,10^. The genome size of coronaviruses ranges between 26 and 32 kilobases^11^. The viruses have characteristic club-shaped spikes projected from their surface, and the surface morphology of the viruses resembles a solar corona^12^. The viruses can be further categorized into gamma, beta, delta and alpha coronaviruses^13^. Beta and alpha coronaviruses originate from bats, while gamma and delta coronaviruses spread among birds and pigs^14^. COVID-19 is a member of beta category, which is associated with severe diseases. The genome structure of COVID-19 is 96% similar to that of bat coronaviruses^15,16^. It is still not clear about the exact route of the virus jump from bats to humans.

COVID-19 had a profound impact on global social and economic development^17^. It caused severe demographic changes and extremely high unemployment rates with many economic activities being halted. This extraordinary event brought about some unintended consequences such as the violation of international law on the settlement of refugees due to border closure^18^.

Different governments and health officials have introduced various preventive measures to curb the COVID-19 virus spread, including hand sanitizers, gloves, masks, social distance, and geographical closure^17^. Although the geographical closure led to temporary urban air quality improvement, lockdown of towns, cities, states, and countries causes severe damage to the well-being and economic growth of society in a broad sense, and the virus poses a major threat to the international healthcare system^19^. The unknown nature about the peak of virus spread makes the decision of lockdown or closure a difficult task to plan in advance. This calls for an accurate early forecasting model for the ongoing spread of COVID-19 virus.

## Literature review

Many studies have been carried out on the epidemic investigation of COVID-19 spread. The first category of studies is a pure statistical analysis. Important epidemic parameters were estimated^20,21^, including basic reproduction number^22^, doubling time^23^ and serial interval^24^. In addition, some advanced models were developed in handling untraced contacts^25^, undetected international cases^26^, and actual infected cases^27^. Statistical reasoning^28,29^ and stochastic simulation^30,31^ were also explored by a few researchers.

The second group of investigations was based on dynamic modelling. Susceptible exposed infectious recovered model (SEIR) was used in assessing various measures in the COVID-19 outbreak^32–35^. Furthermore, it was utilized in investigating the effect of lockdown^36^, transmission process^37^, transmission risk^32^, and the effect of quarantine^32^. The SEIR model with time delays was also developed for studying the period of incubation and recovery^38,39^.

Richards^40^ developed a flexible growth function for empirical use in the context of plant data based on von Bertalanffy’s growth function^41^, which was originally designed for animals. This model was later used for fitting the single-phase outbreaks of severe acute respiratory syndrome (SARS) in Hong Kong^42^ and Taiwan^43^ as well as a multi-phase outbreak of SARS in Toronto^44^. The Georgia State group^45^ recently studied short-term forecasts of the COVID-19 epidemic in Guangdong and Zhejiang, China between February 13 and 23, 2020 via a generalized logistic growth model^46^, the Richards model, and a sub-epidemic model^47^. As an extension to a logistic growth model, the Richards mode can be described by a single differential equation:

1 $$ \frac{dP\left( t \right)}{{dt}} = rP\left( t \right)\left[ {1 - \left( {\frac{P\left( t \right)}{K}} \right)^{a} } \right], $$

where P(t) represents the cumulative number of infected cases at time point *t*, *r* denotes a growth rate, *K* refers to the maximum asymptote, and *a* is a scaling parameter. One solution of Eq. (1) is

2 $$ P\left( t \right) = \frac{K}{{\left[ {1 + e^{{ - r\left( {t - t_{0} } \right)}} } \right]^{\frac{1}{a}} }}, $$

where $t_{0}$ is the time value of the sigmoid’s midpoint. When *a* = 1, this model is degenerated into a simple logistic growth mode with three parameters [*K*, *r*, $t_{0}$]. Equation (2) represents a four-parameter model [*a*, *K*, *r*, $t_{0}$] and other variations of the Richards model could consist of up to 6 parameters. In this paper, our comparison and discussion are limited to Eq. (2).

Although there have been many recent studies with respect to the COVID-19 virus spread, an accurate forecasting model for the virus spread based on data at a very early time point is still elusive. Such a model is crucial to a decision-making process for strategic plans to achieve a balance between reduction in life loss and avoidance of economic crisis due to lockdown. In this paper, we develop a new recursive bifurcation model, apply it to the recent data in two countries (South Korea and Germany), and compare it with a simple logistic growth model and the Richards model in the context of COVID-19 virus spread.

The rest of this paper is organized as follows. In “Recursive bifurcation model”, a recurve bifurcation model is introduced to model the COVID-19 spread. A bifurcation analysis is given in “Bifurcation analysis of COVID-19 virus spread” on the data of infected population in South Korea. “Early forecasting of COVID-19 virus spread” describes the forecasting of COVID-19 virus spread based on our model and a comparative study with two existing models, followed by some concluding remarks in “Conclusions”.

## Recursive bifurcation model

**Figure 1:**
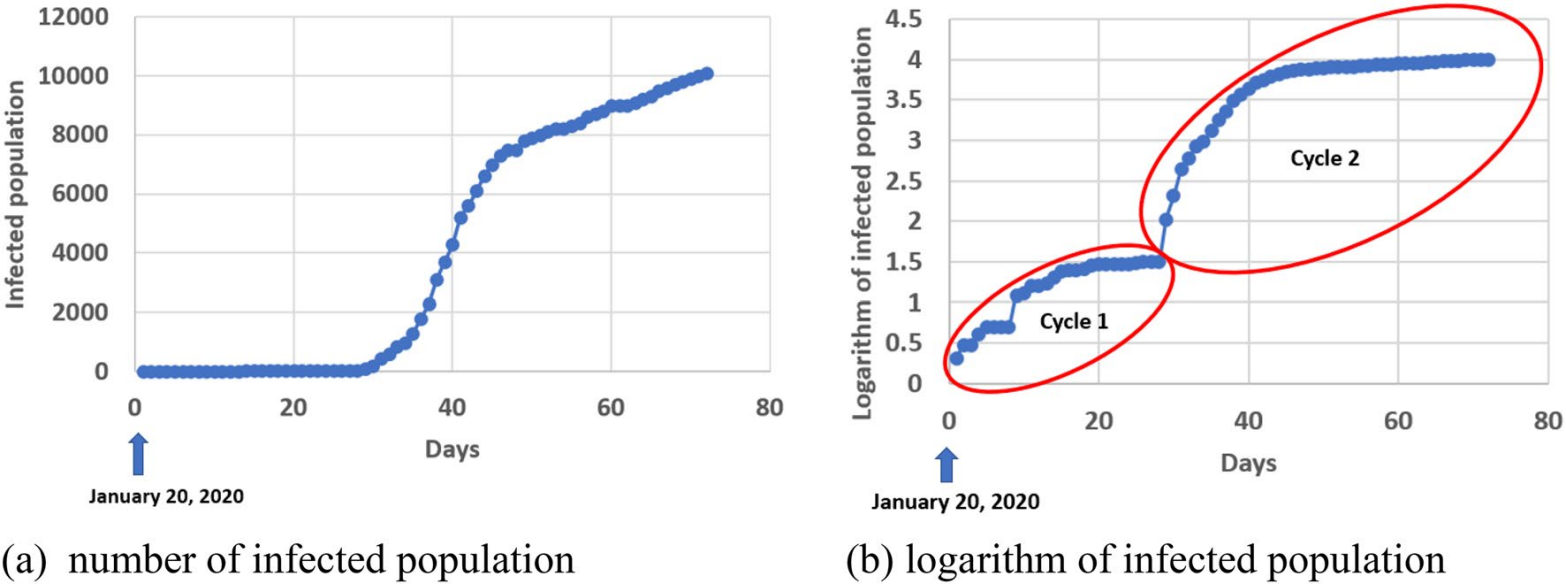
The number of infected population in South Korea as of April 5, 2020.

In this paper, we focus on the cumulative number of infected population, which is an important metric to measure the extent of the COVID-19 spread in different countries. Although the infected population in most countries follows a pattern of a logistic or sigmoid function, the logarithm of the infected population may provide more information, as shown in Fig. 1b.

**Figure 2:**
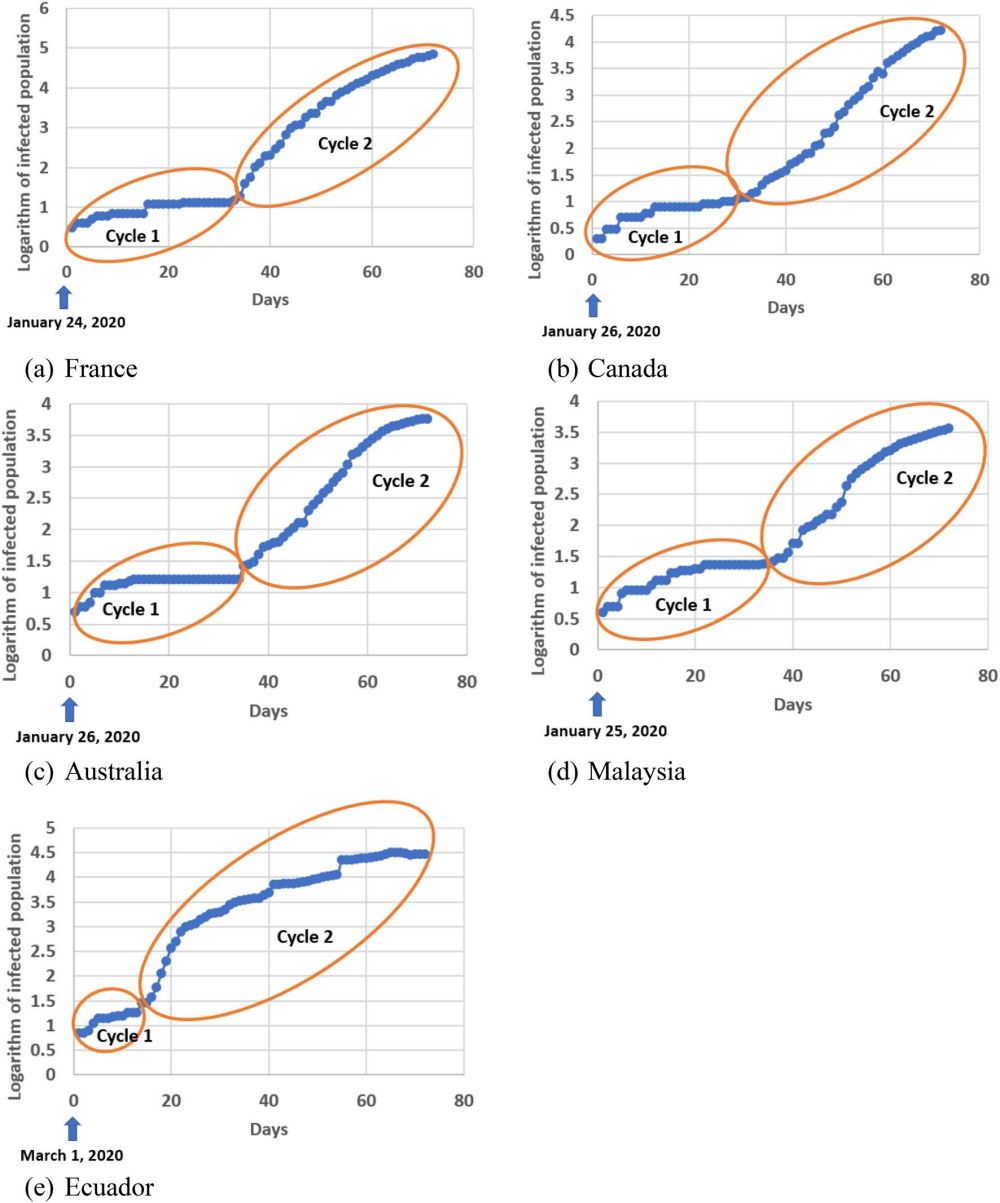
A bifurcation pattern of the infected population of COVID-19 virus spread in five countries.

The countries, in which a bifurcation pattern occurred, include South Korea, Germany, United States, France, Canada, Australia, Malaysia, and Ecuador. Figure 2 shows the pattern in the last 5 countries of the list. The detailed information of Germany and United States data are given in “Early forecasting of COVID-19 virus spread”. By utilizing the bifurcation, we can find out the intrinsic parameters (such as growth rate) in cycle 1 and apply those parameters in the prediction for cycle 2 or beyond. More importantly, the bifurcation model performs better than the Richards model in the early forecasting of COVID-19 virus spread. A more detailed discussion about this improvement is provided in “Early forecasting of COVID-19 virus spread”.

Following the above idea, we introduce a recursive Tanh function to describe the cumulative number of infected population within each cycle of an entire virus spread process:

3 $$ log\left( {P + 1} \right) - log\left( {P_{i - 1} + 1} \right) = \left( {log\left( {P_{i} + 1} \right) - log\left( {P_{i - 1} + 1} \right)} \right)\left[ {\frac{2}{{1 + e^{{ - 2r_{i} \left( {D - D_{i - 1} } \right)}} }} - 1} \right], $$

where *i* refers to the *i*-th cycle, *P* is the cumulative number of infected population at any time point in the *i*-th cycle, *D* represents the number of days since the initiation of virus spread, $P_{i - 1}$ stands for the cumulative number of infected population at the end of the (*i − *1)-th cycle, $r_{i}$ is the spread rate in the *i*-th cycle, and $D_{i - 1}$ refers to the number of days at the end of the (*i − *1)-th cycle. The purpose of adding 1 in the logarithm calculation is to avoid an infinity caused by the case where *P* = 0.

Note that Eq. (3) is not strictly a recursive formula in a conventional sense. The reason for us to call it as a recursive one is that Eq. (3) should be recursively solved starting from cycle 1 toward cycle *n*, if *n* is the last cycle for the virus spread. When *n* = 1, this equation is degenerated to a regular Tanh function:

4 $$ log\left( {P + 1} \right) = log\left( {P_{1} + 1} \right)\left[ {\frac{2}{{1 + e^{{ - 2r_{1} D}} }} - 1} \right]. $$

## Bifurcation analysis of COVID-19 virus spread

In order to validate Eq. (3) for the analysis of COVID-19 virus spread, we have to select a complete virus spread process. Among all the countries, South Korea seems to be the best choice for this validation because the country provides reasonably reliable data and the virus spread in that country has been stabilized.

$r_{i}$ in Eq. (3) represents an intrinsic attribute of the virus spread rate. It can be estimated by a linear least-squares fit of the following linear equation in a parameter space:

5a $$ r_{i} \left( {D - D_{i - 1} } \right) = - 0.5ln\left[ {\frac{2}{{1 + \frac{{log\left( {P + 1} \right) - log\left( {P_{i - 1} + 1} \right)}}{{log\left( {P_{i} + 1} \right) - log\left( {P_{i - 1} + 1} \right)}}}} - 1} \right], $$

5b $$ r_{i} X = W, $$

where $$X = D - D_{i}$$ and $$ W = - 0.5 ln \left[ {\frac{2}{{1 + \frac{{log\left( {P + 1} \right) - log\left( {P_{i - 1} + 1} \right)}}{{log\left( {P_{i} + 1} \right) - log\left( {P_{i - 1} + 1} \right)}}}} - 1} \right]$$.

**Figure 3:**
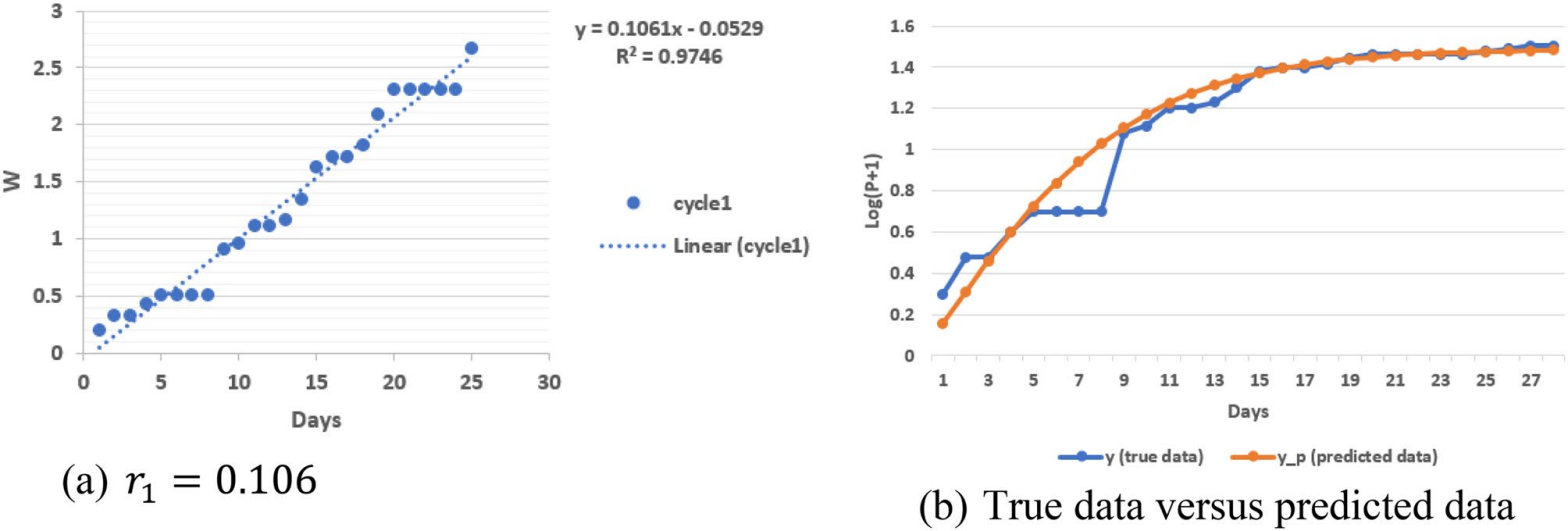
Determination of virus spread rate with South Korea data in cycle 1.

Figure 3a shows the result of determining the virus spread rate, $r_{1}$. By using this *r* value, we predict the infected population, $y_{p}$, which is very close to the true data, *y*, as shown in Fig. 3b.

**Figure 4:**
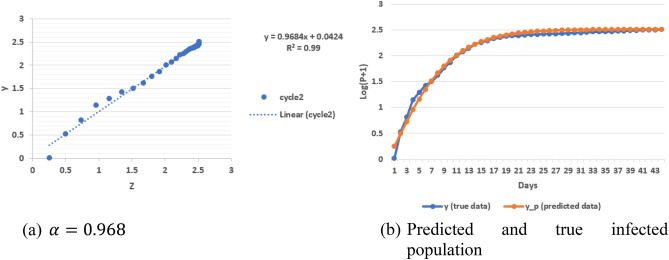
Analysis of virus spread with South Korea data in cycle 2.

Furthermore, by using $r_{1}$ in cycle 2 of South Korea data, we want to validate whether Eq. (3) is still valid by introducing Eq. (6), and $\alpha$ should be unity (Fig. 4):

6a $$ log\left( {P + 1} \right) - log\left( {P_{i - 1} + 1} \right) = \alpha \left( {log\left( {P_{i} + 1} \right) - log\left( {P_{i - 1} + 1} \right)} \right)\left[ {\frac{2}{{1 + e^{{ - 2r_{i} \left( {D - D_{i - 1} } \right)}} }} - 1} \right], $$

6b $$ y = \alpha Z, $$

where $$y = log\left( {P + 1} \right) - log\left( {P_{i - 1} + 1} \right)$$ and $$Z = \left( {log\left( {P_{i} + 1} \right) - log\left( {P_{i - 1} + 1} \right)} \right)\left[ {\frac{2}{{1 + e^{{ - 2r_{i} \left( {D - D_{i - 1} } \right)}} }} - 1} \right]$$.

Based on Fig. 4, $\alpha = 0.968$, which is very close to unity. This indirectly indicates the correctness of Eq. (3) for cycle 2 with growth rate, $r_{1}$, from cycle 1. The bifurcation in Fig. 1a is easy to identify visually. An automatic algorithm can be created on the basis of discontinuity of tangential direction with a traverse on the curve. Since it is not the main focus of this paper, we do not explore this aspect any further herein.

## Early forecasting of COVID-19 virus spread

Based on the model in “Bifurcation analysis of COVID-19 virus spread”, we design an algorithm for early forecasting of COVID-19 virus in South Korea and Germany, as given in Table 1. Since the infected population has recently been stabilized in these two countries, it is then possible to validate the accuracy of this early forecasting model.

**Table 1:** An algorithm for early forecasting of COVID-19 virus spread.

Step 1	Determine the virus spread rate in cycle 1, $r_{1}$, based on a least-squares fitting of Eq. (5)
Step 2	Recursively analyze the infected population in cycles 2 through *n − *1
Step 3	Let the virus spread rate in cycle *n*,$\hat{r}_{n} = r_{n - 1}$
Step 4	Estimate an initial value of the logarithm of infected population in cycle *n* by a linear least-squares fitting of $\hat{\beta }_{n}$ in Eq. (7)
Step 5	Determine $\beta_{n}$, $\theta_{n}$ and $D_{n}$ by using a nonlinear Levenberg–Marquart least-squares fitting through Eq. (8)
Step 6	Predict the future infected population based on Eq. (8)

We first use the following formula to estimate $\hat{\beta }_{n}$ through a linear least-squares fit:

7a $$ log\left( {P + 1} \right) - log\left( {P_{n - 1} + 1} \right) = \hat{\beta }_{n} \left[ {\frac{2}{{1 + e^{{ - 2r_{n} \left( {D - D_{n - 1} } \right)}} }} - 1} \right], $$

7a $$ y = \hat{\beta }_{n} { }Z, $$

where $$y = log\left( {P + 1} \right) - log\left( {P_{n - 1} + 1} \right)$$ and $$Z = \left[ {\frac{2}{{1 + e^{{ - 2r_{n} \left( {D - D_{n - 1} } \right)}} }} - 1} \right]$$.

Nonlinear Levenberg-Marquart least-squares fitting^48^ is computed to determine three unknown parameters ($\beta_{n}$, $\theta_{n}\,and\,D_{n}$) simultaneously in the following equation for future forecasting:

8 $$ log\left( {P + 1} \right) = \beta_{n} \left[ {\frac{2}{{1 + \left( {e^{{ - 2r_{n - 1} \left( {D - D_{n} } \right)}} } \right)^{{\theta_{n} }} }} - 1} \right] + log\left( {P_{n - 1} + 1} \right), $$

where $\theta_{n}$ is an extra parameter, which plays a role of slope control. Once $\beta_{n}$, $\theta_{n}$ and $D_{n}$ are determined, Eq. (8) can be used to predict the values of infected population at future time points.

**Figure 5:**
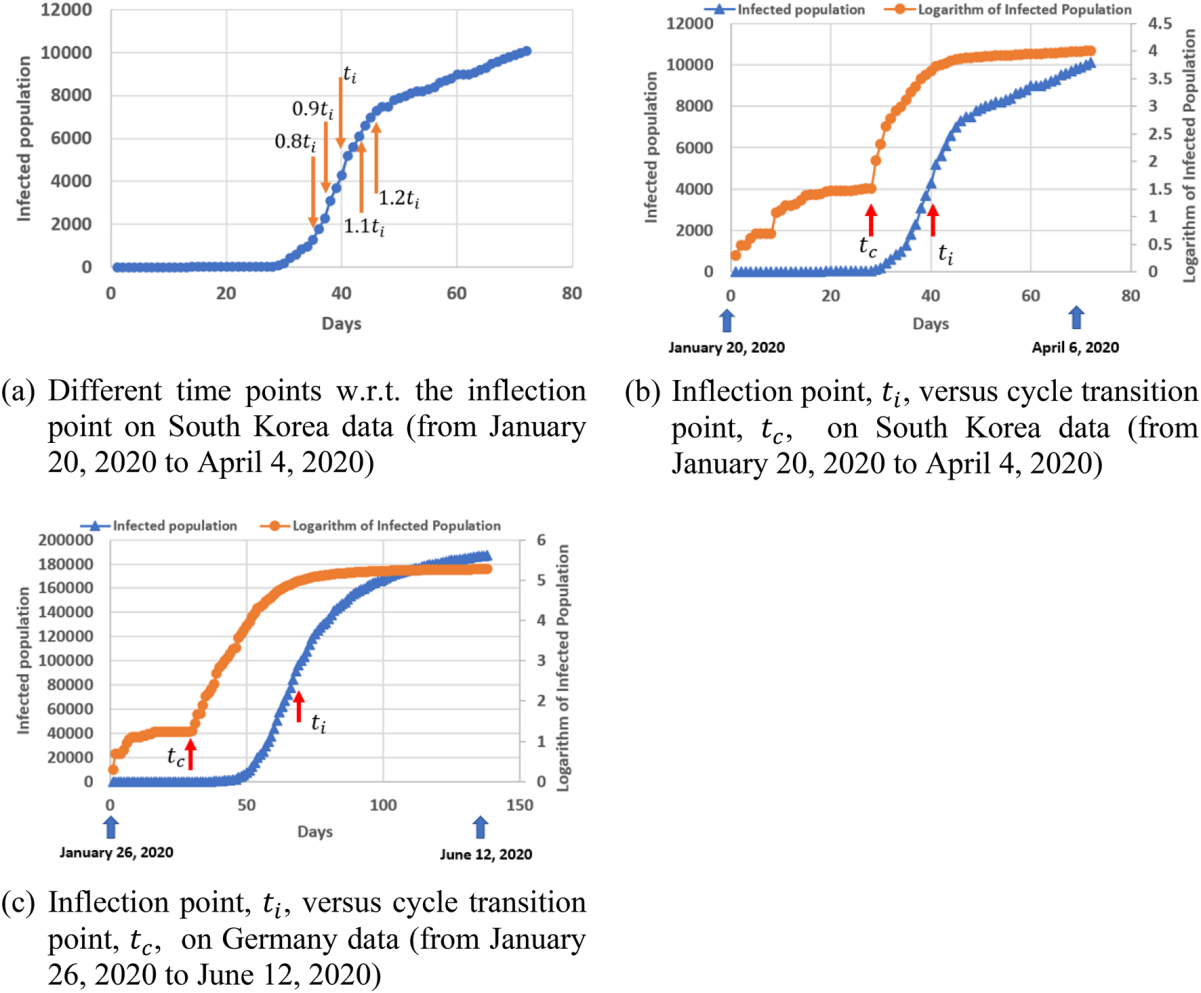
Notations for early forecast on future infected population.

Figure 5a defines several different time points to investigate the performance of early forecast on the “future” infected population. Here, the “future” is termed only in a sense with respect to a selected time point (i.e., a reference point after which the “future” is factitiously defined) even though we already have the true infected population data for a period after that time point. $t_{i}$ refers to the time value of the inflection point. $0.9t_{i}$, $0.8t_{i}$, $...,$ and 0 equally divide the range [0, $t_{i}$] into ten intervals. We use a similar interval for time values that are greater than $t_{i}$: $1.1t_{i}$, $1.2t_{i}$, $...,$ and $mt_{i}$, where $m$ could be any real number and should be greater than unity.

It is shown in Fig. 5b that the infection point, $t_{i}$, appeared 12 days later than the cycle transition point, $t_{c}$, between cycles 1 and 2 on South Korea data; a similar pattern was also observed in U.S., Germany and the countries listed in Fig. 2. This provides a better opportunity for our bifurcation method to produce an early forecast, compared to existing methods such as the Richards method, which generates a reasonably good prediction only at a time point after the inflection point because the right part of a curve after the inflection point can’t normally be mirrored from the left part of the curve before the inflection point. In the case of Germany data (Fig. 5c), $t_{c}$ appeared 38 days earlier than $t_{i}$. This provides an opportunity for our bifurcation method to utilize the cycle information for early forecast on the COVID virus spread.

**Figure 6:**
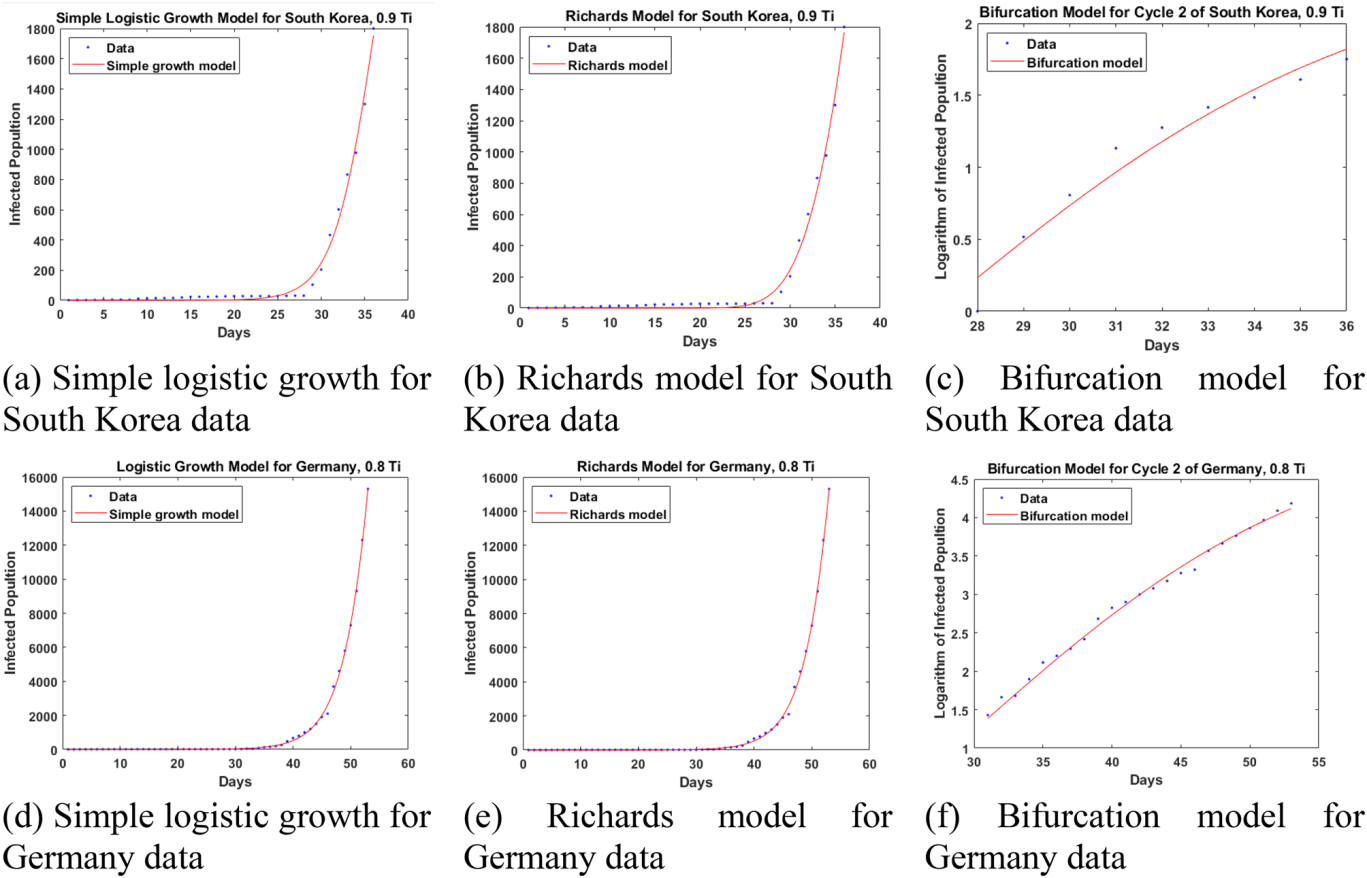
Curve fitting of two-country data at their respective reference time points with three different models.

Tables 2 and 3 are a comparison in the 95 percent confidence interval of prediction errors of three models at a reference time point defined in Fig. 5a. The second column of these tables contains a mean value and 95 percent confidence bounds in a pair of parentheses. In general, our bifurcation model performs relatively better for an early forecast at 0.8 $T_{i}$ or 0.9 $T_{i}$. The forecast time point was selected to a date of writing this paper. Between the Richards model and a simple logistic growth model, the former is better in terms of relative error in one out of two cases. Since there are at least 4 parameters [*a*, *K*, *r*, $t_{0}$] in the Richards model, closely-estimated start values are needed in nonlinear least-squares fitting. Figure 6 shows the curve fitting of two-country data based on the three models used in this paper. The parameters associated with each model are given in Table 4.

**Table 2:** A comparison in infected population prediction at 3.55 $$T_{i} $$ based on South Korea data at 0.9 $$T_{i}$$.

Model	95 percent confidence interval of infected population predicted for 3.55 $T_{i}$ time point	True infected population at 3.55 $T_{i}$	Absolution relative error of mean value for forecast at 3.55 $T_{i}$ (%)
Simple logistic growth	3560 (2126, 4995)	12,051	70.5
Richards	18,070 (− 115,497, 151,697)	12,051	49.9
Our bifurcation	9488 (4468, 20,144)	12,051	21.3

General information:

Start time: January 20, 2020; Cycle transition time: $T_{c} =$ 28 days.

Inflection time point: $T_{i} =$ 40 days; Reference time point: 0.9 $T_{i} =$ 36 days.

Forecast time point: 3.55 $T_{i} =$ 142 days (June 12, 2020).

**Table 3:** A comparison in infected population prediction at 2.0 $$T_{i} $$ based on Germany data at 0.8 $$T_{i}$$.

Model	95 percent confidence interval of infected population predicted for 2.0 $T_{i}$ time point	True infected population at 2.0 $T_{i}$	Absolution relative error of mean value for forecast at 2.0 $T_{i}$ (%)
Simple logistic growth	109,400 (40,070, 178,800)	187,226	41.6
Richards	43,340 (-68,990, 155,700)	187,226	76.8
Our bifurcation	178,373 (63,316, 502,508)	187,226	4.7

General information:

Start time: January 26, 2020; Cycle transition time: $T_{c} =$ 30 days.

Inflection time point: $T_{i} =$ 68 days; Reference time point: 0.8 $T_{i} =$ 54 days.

Forecast time point: 2.0 $T_{i} =$ 138 days (June 12, 2020).

**Table 4:** Model parameters associated with the fitting in Fig. 6.

Model	Country	95 percent confidence bounds of parameters
Simple logistic growth	South Korea	*K:* 3560 (2126, 4995) *r:* 0.4268 (0.3563, 0.4974) $$t_{0} : $$ 36.08 (34.31, 37.84)
	Germany	*K:* 6.633e+04 (6.048e+04, 7.219+04) *r:* 0.2607 (0.2465, 0.2749) $t_{0} :$ 57.8 (57.09, 58.51)
Richards model	South Korea	*a:* 0.05037 (− 1.333, 1.434) *K:* 1.807e+04 (− 1.155e+05, 1.517e+05) *r*: 0.1108 (− 0.3716, 0.5933) $t_{0} :$ 17.19 (− 297.2, 331.5)
	Germany	*a:* 0.1296 (− 0.2247, 0.4839) *K:* 1.441e+05 (3.597e+04, 2.521e+05) *r:* 0.09903 (0.03072, 0.1673) $t_{0} :$ 42.11 (6.516, 77.7)
Bifurcation model	South Korea	$\beta_{n} :$ 2.48 (2.144, 2.815) $\theta_{n} :$ 1 (fixed at bound) $D_{n}$: 27.14 (26.32, 27.95)
	Germany	$\beta_{n} :$ 4.023 (3.573, 4.473) $\theta_{n} :$ 0.3635 (0.3097, 0.4174) $D_{n}$: 30 (fixed at bound)

Note that for the data from South Korea and Germany, there is no multi-stage pattern if the simple logistic growth model or the Richards model is used. Only through the special treatment in our bifurcation model, did a two-stage pattern appear, allowing a more accurate forecast at an early time point (such as 0.8 $$T_{i} $$ or 0.9 $$T_{i}$$) on the infected population growth at a later time point (for example, 2.0 $$T_{i}$$).

The importance of our model is supported by the results in Tables 2 and 3, where our model performs significantly better than the existing models in terms of early forecast on the growth of COVID-19 virus spread. Consequently, our model has a potential to be used in decision making for the events of virus spread in the future.

**Figure 7:**
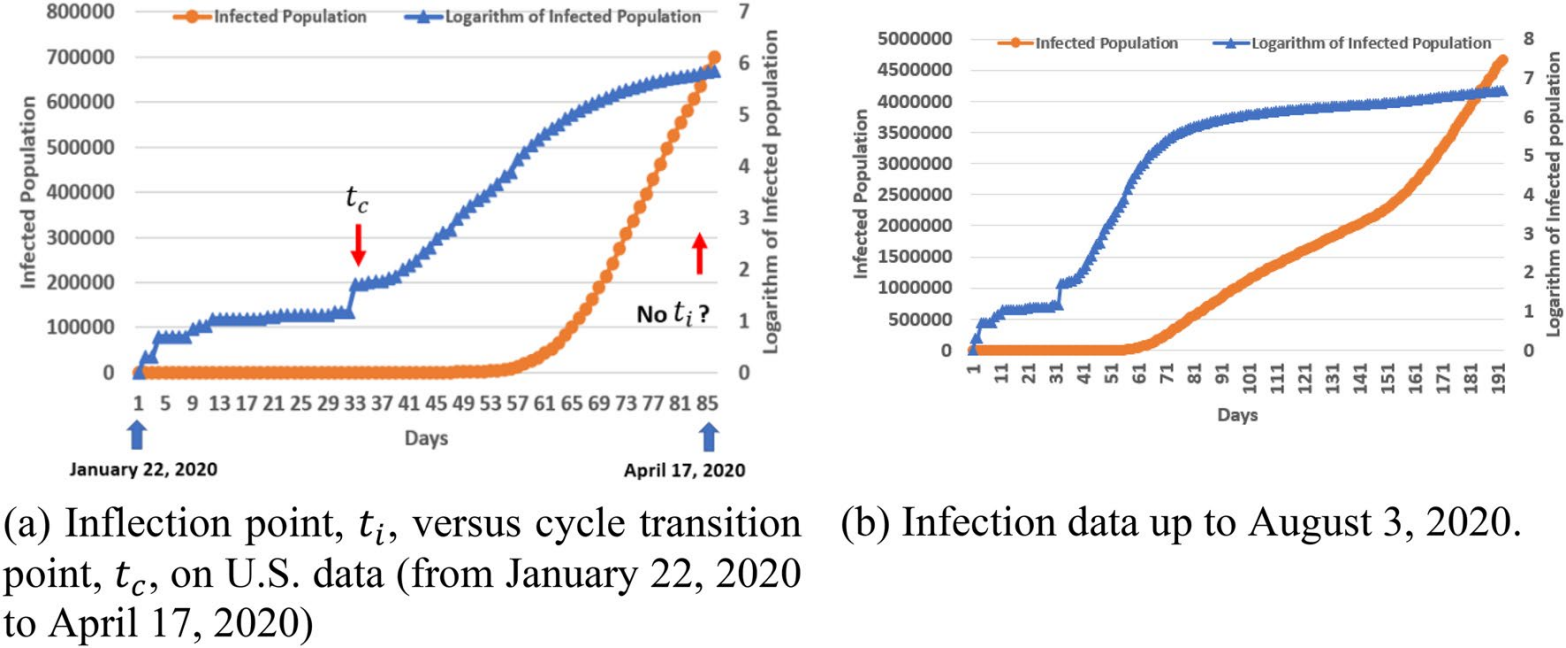
Infected population data of COVID-19 in United States.

The data from United States presents a challenge to our approach and also reflects a limitation of the method. As shown in Fig. 7a, for the period from January 22, 2020 to April 17, 2020, there was no inflection point and our cycle transition point appeared very early (Day 33). This case indicates that the cycle transition point appeared at least 52 days earlier than the inflection point. However, since the inflection point did not appear even on August 3, 2020 (Fig. 7b), it is difficult to validate the accuracy of any early forecasting models on U.S. data at the time of writing this paper. It is still elusive how to design and evaluate an early forecasting model when the entire virus spread history is at its early stage without the occurrence of its inflection point. This will be a future research topic.

## Conclusions

In this paper, we propose a recursive bifurcation approach for early forecasting of COVID-19 virus spread. An algorithm is developed to predict the future infected population based on ongoing existing data as of June 14, 2020. Numerical analyses were conducted in comparison with two existing models (a logistic growth model and a Richards model). The results indicate that our bifurcation model performs relatively better than the two existing models at 0.8 $$T_{i} $$ or 0.9 $$T_{i}$$ time point, where $$T_{i} $$ refers to the inflection point of an infected population-time curve. We presented an important observation in which the cycle transition time point, $$T_{c}$$, appeared much earlier than $$T_{i}$$. This allows our bifurcation model to perform well in the early forecasting of COVID-19 virus spread in South Korea and Germany. However, the ongoing infection spread in United States presents a challenge to our model. It will be a future research topic on how to evaluate the forecasting of COVID-19 infection spread when its inflection point has not occurred as of August 3, 2020.

## Data Availability

The dataset of this article has been published at Harvard Dataverse with the following link: Shen, Julia, 2020, “Recursive Bifurcation Model for COVID-19 Virus Spread”, 10.7910/DVN/PVCPWM, Harvard Dataverse, V1.
